# Genetic and epigenetic susceptibility of airway inflammation to PM_2.5_ in school children: new insights from quantile regression

**DOI:** 10.1186/s12940-017-0285-6

**Published:** 2017-08-18

**Authors:** Yue Zhang, Muhammad T. Salam, Kiros Berhane, Sandrah P. Eckel, Edward B. Rappaport, William S. Linn, Rima Habre, Theresa M. Bastain, Frank D. Gilliland

**Affiliations:** 1 Division of Epidemiology, Department of Internal Medicine, University of Utah, 295 Chipeta Way, Salt Lake City, UT 84018 USA; 2 Department of Family and Preventive Medicine, University of Utah, Salt Lake City, UT USA; 3 Veteran Affairs Salt Lake City Health Care System, Salt Lake City, UT USA; 4 Department of Preventive Medicine, University of Southern California, Los Angeles, CA USA; 5 Department of Psychiatry, Kern Medical, Bakersfield, CA USA

**Keywords:** Exhaled nitric oxide, PM_2.5_, Asthma, Inducible nitric oxide synthase, Haplotype, Methylation

## Abstract

**Background:**

The fractional concentration of exhaled nitric oxide (FeNO) is a biomarker of airway inflammation that has proved to be useful in investigations of genetic and epigenetic airway susceptibility to ambient air pollutants. For example, susceptibility to airway inflammation from exposure to particulate matter with aerodynamic diameter < =2.5 μm (PM_2.5_) varies by haplotypes and promoter region methylation in inducible nitric oxide synthase (iNOS encoded by NOS2). We hypothesized that PM2.5 susceptibility associated with these epigenetic and genetic variants may be greater in children with high FeNO from inflamed airways. In this study, we investigated genetic and epigenetic susceptibility to airborne particulate matter by examining whether the joint effects of PM2.5, *NOS2* haplotypes and iNOS promoter methylation significantly vary across the distribution of FeNO in school children.

**Methods:**

The study included 940 school children in the southern California Children’s Health Study who provided concurrent buccal samples and FeNO measurements. We used quantile regression to examine susceptibility by estimating the quantile-specific joint effects of PM_2.5_, *NOS2* haplotype and methylation on FeNO.

**Results:**

We discovered striking differences in susceptibility to PM_2.5_ in school children. The joint effects of short-term PM_2.5_ exposure, *NOS2* haplotypes and methylation across the FeNO distribution were significantly larger in the upper tail of the FeNO distribution, with little association in its lower tail, especially among children with asthma and Hispanic white children.

**Conclusion:**

School-aged children with higher FeNO have greater genetic and epigenetic susceptibility to PM_2.5_, highlighting the importance of investigating effects across the entire distribution of FeNO.

**Electronic supplementary material:**

The online version of this article (doi:10.1186/s12940-017-0285-6) contains supplementary material, which is available to authorized users.

## Background

The fractional concentration of exhaled nitric oxide (FeNO) is a quantitative, noninvasive, easily measurable biomarker related to airway inflammation, responsive to adverse effects of air pollution on respiratory health [1–4]. Although the mechanisms by which exposure to air pollutants causes airway inflammation are not yet fully understood, high levels of NO are ultimately produced from bronchial epithelial cells and other resident cell types by the induction of inducible nitric oxide synthase (iNOS, encoded by *NOS2*).

In recent years, extensive research has shown that elevated ambient-background and traffic-related air pollutants are both associated with higher FeNO in healthy subjects and in patients with asthma and COPD [1, 5–9]. In the southern California Children’s Health Study (CHS), we reported that both long- and short-term exposures to ambient fine particulate matter (PM_2.5_) were associated with higher FeNO in school children [10, 11]. In terms of genetic determinants of FeNO, variants in *NOS2* on chromosome 17 have been shown to be significantly associated with FeNO in both children and adults [12–16]. Data from CHS also showed that promoter haplotypes in *NOS2* had affected respiratory health and were significantly associated with FeNO, asthma incidence and pulmonary function development in school children [17]. Building on those findings, we further found that *NOS2* genetic and epigenetic variations and short-term PM_2.5_ exposure jointly affected FeNO level [18]. This novel finding suggested that the susceptibility of airway inflammation to short-term exposure to PM_2.5_ is modified by haplotype and iNOS promoter methylation [18].

Because children with asthma and other conditions associated with inflamed airways show larger responses to particulate air pollutants, we hypothesized that PM_2.5_ susceptibility associated with these epigenetic and genetic variants may be greater in children with high FeNO from inflamed airways. All the studies above were performed using standard “mean-based” linear regression, which reports the change in mean outcome for a given change in exposure. This approach assumes that effects of risk factors on FeNO only shift the location of the FeNO distribution; thus it may obscure heterogeneity in the effects by failing to capture associations that differ across the FeNO distribution. A different analytic method is needed to address our hypothesis that PM2.5 susceptibility for airway inflammation (as measured by FeNO) associated with these epigenetic and genetic variants is greater in children with existing airway inflammation and high FeNO. For example, in a novel application of quantile regression, asthma, current rhinitis, gender and race/ethnicity were found to be associated with larger increases in the upper tails of the FeNO distribution suggesting that inflamed airways show enhanced difference for these factors [19]. In the present study, we used quantile regression methods to examine data from the CHS on air pollution exposures, genetic and epigenetic data to evaluate variation in their joint effects on FeNO across its distribution, and determined whether the joint effects varied by asthma status, race/ethnicity, gender or allergy.

## Methods

We evaluated the population of 940 non-Hispanic white and Hispanic white children analyzed previously [18, 20]. Each child had FeNO measurement and buccal sample collection on the same day in one of three consecutive school years (2004–2005, 2005–2006, 2006–2007). The institutional review board at the University of Southern California approved the study protocol. All subjects assented and their parents or legal guardians consented. The brief description of collected data in the study is provided in the following subsections. More detailed information on methods for data collection could be found in our previous publications [18, 20].

### FeNO measurement

In years 2004–2005 and 2005–2006, offline FeNO samples were collected in bags at 100 ml/s expiratory flow following American Thoracic Society (ATS) guidelines [21]. In 2006–2007, online FeNO was measured using EcoMedics CLD-88-SP analyzers at 50 ml/s expiratory flow following ATS guidelines [21]. In our previous publication, we used 361 school children in the CHS study who had FeNO measured using both online and offline techniques to develop and validate a model predicting online FeNO from offline FeNO (adjusted *R* ^*2*^ = 0.94) [22]. In this study, we used predicted online FeNO for children measured in the first two years, and online FeNO for children measured in the third year.

### PM_2.5_

PM_2.5_ data were obtained from central monitoring sites in each study community operated by local air pollution agencies in conformance with US Environmental Protection Agency (EPA) requirements. Each community contained a single central-site monitor. At each monitoring site, 24-h average measurements PM_2.5_ were obtained daily or every third day. When pollution data were not available for certain days, the gaps were filled using data from nearby monitors provided that the monitors were not more than 7 km apart. Daily 24-h averages were used to calculate cumulative average exposure levels 7 days prior to the FeNO test date, referred to as “short-term PM_2.5_ exposure” hereafter.

### Haplotype selection and methylation in NOS2 promoter

We selected 7 SNPs in *NOS2* promoter region, which represents a minimum set of haplotype-tagging SNPs (htSNPs) with MAFs ≥0.05 to explain >90% of haplotype diversity (R^2^h ≥ 0.90). The order of selected SNP in *NOS2* promoter haplotypes is rs4795080-rs2779253-rs1889022-rs10853181-rs2531866-rs1014025-rs25318723. In this study, the primary *NOS2* promoter haplotype of interest is h1000010 [H1], which is one of most common haplotype variants and shown to impact the mean FeNO level jointly with short-term PM_2.5_ and methylation in previous publication [18]. Other common haplotype variants: h0111101 [H2], h0000000 [H3] and h0000010 [H4] were also explored separately as secondary haplotype of interest. The haplotype frequencies were estimated by using an SAS macro code available with the TagSNPs program. This haplotype estimation technique provides the maximum likelihood estimates of the haplotype frequencies assuming Hardy-Weinberg equilibrium.

The methylation site was 8091 bp down-stream to the nearest *NOS2* promoter SNP (rs4795080). The DNA methylation analyses were performed by bisulfite-PCR with appropriate quality control. Laboratory personnel performing DNA methylation analyses were blinded to study subject information. Bisulfite conversion of 1 μg of genomic DNA extracted from buccal mucosal cells were performed with the EZ-96 DNA Methylation Gold Kit (Zymo Research, Orange, CA), according to the manufacturer’s recommended protocol. Final elution was performed with 40 μL M-elution buffer. Bisulfite-converted DNA was stored at −70 °C until further use. Pyrosequencing assays were performed using the HotMaster Mix (Eppendorf Hamburg, Germany) and the PSQ HS96 Pyrosequencing System (Biotage AB, Uppsala, Sweden).

### Other covariates

Race/ethnicity, annual family income, parental education, exposure to secondhand tobacco smoke (SHS), asthma history and allergy history were determined from annual written questionnaires completed by the parents. Questionnaires were collected at the school visit during which the child’s buccal cell sample was collected. Height and weight were measured on the day of FeNO testing. Body mass index (BMI) was categorized as underweight, normal, overweight or obese based on the Centers for Disease Control and Prevention growth charts (https://www.cdc.gov/nccdphp/dnpao/growthcharts/resources/sas.htm).

### Statistical methods

Descriptive data analyses were performed to examine characteristics of the study population and to characterize the distribution of FeNO by these factors. A quantile regression (QR) approach [23] was used to examine the joint effects of short-term PM_2.5_ exposure (PM), *NOS2* promoter haplotypes (H) and iNOS promoter methylation (M) on specific percentiles (or quantiles, denoted by τ) of the response variable, natural log-transformed FeNO, as shown below:

$$ {Q}_{logFeNO}\left(\tau \right)={\beta}_0\left(\tau \right)+{\beta}_1\left(\tau \right)\bullet \mathrm{PM}+{\beta}_2\left(\tau \right)\bullet \mathrm{M}+{\beta}_3\left(\tau \right)\bullet \mathrm{H}+{\beta}_4\left(\tau \right)\bullet \mathrm{PM}\bullet \mathrm{M}+{\beta}_5\left(\tau \right)\ \mathrm{PM}\bullet \mathrm{H}+{\beta}_6\left(\tau \right)\bullet \mathrm{H}\bullet \mathrm{M}+{\beta}_7\left(\tau \right)\bullet \mathrm{PM}\bullet \mathrm{H}\bullet \mathrm{M} $$

In the QR model, all regression coefficients are indexed by quantile τ (where, for example, τ = 0.5 corresponds to the median) and *β*_1_(*τ*) − *β*_7_(*τ*) quantify the change in the value of the quantile of log-transformed FeNO, *Q*_*logFeNO*_(*τ*), associated with unit change in the predictor variables PM , M and H. All models were adjusted for the design variables age and community of residence as well as gender, race/ethnicity, asthma, respiratory allergy, parental education, secondhand tobacco smoking, month of FeNO collection and experimental plate (for Pyrosequencing reactions). Furthermore, we use 7 degree freedom likelihood ratio tests to examine whether the joint effects of PM_2.5_, *NOS2* promoter haplotypes, iNOS promoter methylation on FeNO level are modified by variables such as asthma status, race/ethnicity, gender and allergy. We fitted the QR models at selected quantiles ranging from 0.10 to 0.90, with 0.20 increments. Since inference in quantile regression does not rely on the normality assumption, log-transformation of FeNO is not needed in general. However, we modeled log-transformed FeNO because it is more linearly associated with covariates than original scale FeNO, and we also wanted to compare quantile regression results to results from traditional mean regression of log-transformed FeNO. All models were fitted using the *quantreg* package in R (http://cran.r-project.org/) using the method discussed in [23]. Hypothesis tests were performed under a 0.05 significance level and a two-sided alternative.

We also constructed a way to visually present the distortion of FeNO distribution by contrasting the empirically observed FeNO distribution among study cohort with their predicted distribution for a given change in PM_2.5_, haplotype and methylation exposure using the fitted quantile regression model.

## Results

Participants were between 6 and 11 years old (mean = 9.3) and equally divided between boys and girls (Table 1). Most children were non-Hispanic White (64.6%). The geometric mean of FeNO was 11.1 *ppb* (geometric SD = 1.9). Consistent with previous literature, we found that age and history of asthma and respiratory allergy were associated with higher FeNO level (all *P* < 0.001). Gender, BMI, parental education and SHS were not significantly associated with FeNO. Among children with asthma, geometric mean FeNO was 14.8 *ppb* (geometric SD = 2.4), and the FeNO level at 80th percentile was about 4 times the median. There were 62.8% of the children in the study population with short-term PM_2.5_ levels >10 μg/m^3^. The observed variation of short-term PM_2.5_ exposure by community in the study cohort is provided in Additional file 1: Figure S1.

**Table 1:** Summary of Selected Characteristics of CHS FeNO Study Participants

	*N* (%)	Distribution of FeNO						
		Median (IQR)	0.2	0.4	0.6	0.8	Mean (SD)	*P*-values
Age [mean(range)]	9.3 (6.4,11.7)	9.75 (8.4)	6.5	8.5	11.3	17.3	11.1 (1.9)	<0.001
Gender								
Girl	489 (52%)	10.2(7.9)	6.6	8.8	11.5	17	11.2 (1.9)	0.72
Boy	451 (48%)	9.3(8.85)	6.4	8.3	10.8	17.9	11 (2)	
Ethnicity								
Hispanic White	607 (64.6%)	10(8.55)	6.6	8.5	11.9	17.8	11.4 (2)	0.16
Non-Hispanic White	333 (35.4%)	9.5(7.3)	6.3	8.5	10.9	16.5	10.7 (1.9)	
Asthma								
No	807 (85.9%)	9.6(7.75)	6.4	8.4	11.1	16.3	10.6 (1.8)	<0.001
Yes	133 (14.1%)	10.7(22)	7	8.9	14.2	36.4	14.8 (2.4)	
History of Respiratory Allergy								
No	418 (44.5%)	9.5(5.975)	6.6	8.4	10.5	14.7	10.2 (1.8)	<0.001
Yes	522 (55.5%)	10.15(10.575)	6.4	8.6	12.8	21.6	11.9 (2)	
Exposure to Secondhand Smoke								
No	909 (96.7%)	9.7(8.4)	6.5	8.5	11.3	17.3	11.1 (1.9)	0.92
Yes	31 (3.3%)	10.8(7.65)	6.5	8.4	12.4	15.2	11 (2)	
Body mass index								
Underweight (< 5th percentile)	11 (1.2%)	13.5(6.3)	7.5	11.2	13.8	14.4	12.5 (2)	0.90
Normal (5th to <85th percentile)	550 (58.5%)	9.7(8.3)	6.6	8.5	11.1	17.1	11 (1.9)	
Overweight (85th to <95th percentile)	183 (19.5%)	9.5(8)	6.4	8.2	10.9	17.8	11.1 (2)	
Obese (≥ 95th percentile)	191 (20.3%)	10(8.35)	6.5	8.7	12.1	18.2	11.3 (2)	
Parental Education								
<12th grade	238 (25.3%)	11.15(8.925)	7.4	9.7	13.2	19.0	12.3 (1.9)	0.08
12th grade	163 (17.3%)	9.6(8.85)	6.1	7.7	11.3	17.5	11 (2.1)	
Some college	302 (32.1%)	9.3(7.375)	6.4	8.1	10.4	16.6	10.9 (2)	
College	125 (13.3%)	9.3(8.5)	6.3	8.4	10.98	16.0	10.6 (1.9)	
Some graduate	112 (11.9%)	9.35(7.45)	6.4	8.0	10.8	16.5	10.2 (1.8)	
Annual family income								
<$15,000	275 (29.3%)	10.7(9.5)	7.2	9.5	12.9	19.4	12.3 (2)	0.003
$15,000 - $49,999	240 (25.5%)	10.35(8.2)	6.5	8.7	12.4	18.3	11.4 (2)	
≥$50,000	425 (45.2%)	9.1(6.9)	6.3	8.1	10.3	15.7	10.3 (1.9)	

Using mean-based regression [18], short-term PM_2.5_ exposure, *NOS2* haplotype and percent iNOS promoter methylation were jointly associated with mean FeNO (three-way interaction *P* < 0.01). Using quantile regression, we further discovered that these associations were not homogenous across the quantiles of FeNO. Significant associations were observed among subjects with the highest FeNO (Additional file 1: Table S1).

**Fig. 1:**
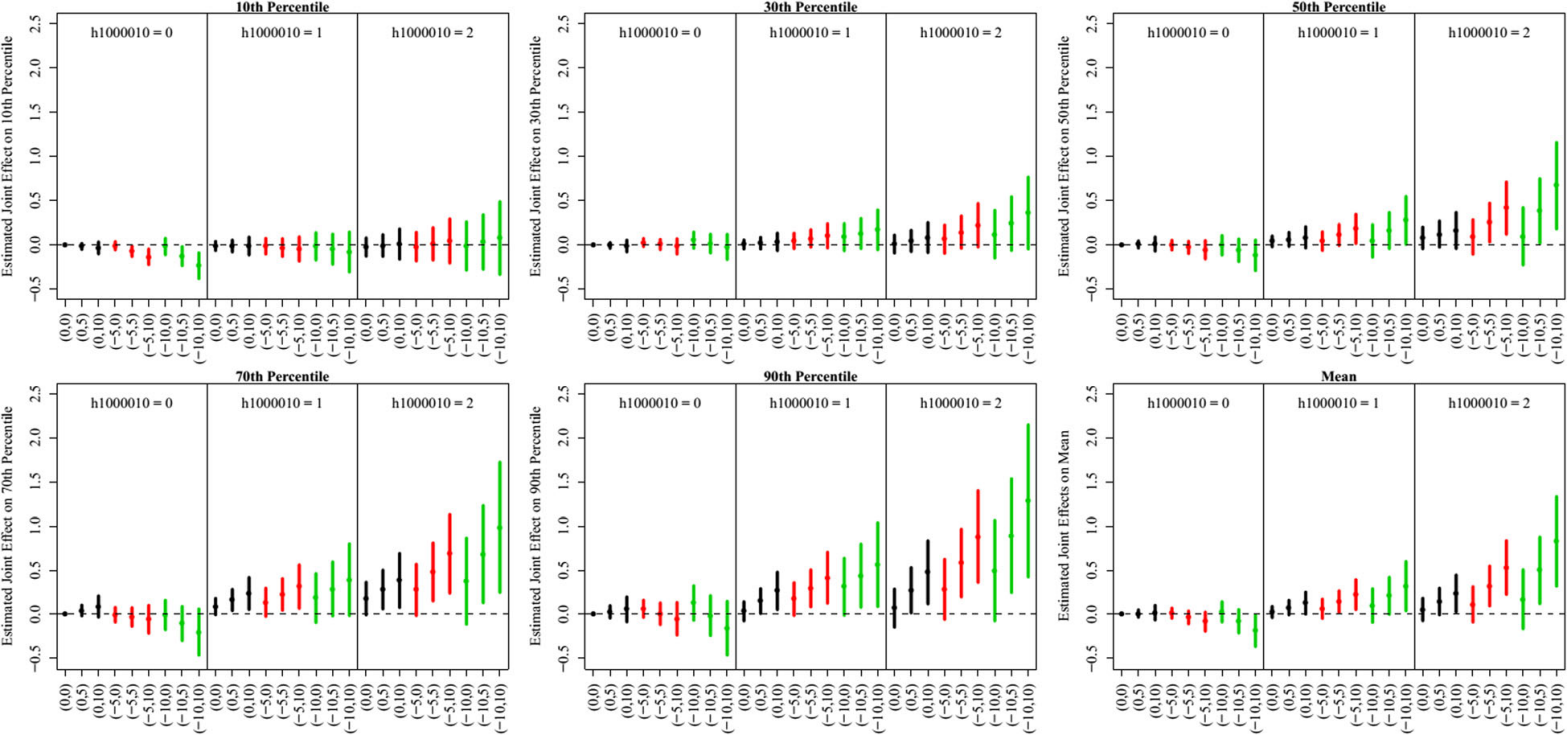
Estimated joint effects of *NOS2* H1 haplotype, iNOS methylation and 7-day average PM_2.5_ exposure across the selected quantiles of FeNO distribution and its mean. In each panel, data are presented by number of H1 haplotype copy. The X-axis shows the combination of levels in methylation (first number in the bracket) and short-term PM_2.5_ exposure (second number in the bracket). Selected methylation levels are population average, 5% and 10% lower than averages, which are indexed by 0,-5 and −10, respectively. Selected PM_2.5_ exposure levels are population average, 5 μg/m^3^ and 10 μg/m^3^ higher PM_2.5_ exposure levels than average, which are indexed by 0, 5 and 10, respectively. The estimated joint effects when methylation levels are at population average, 5% and 10% lower than average are represented by black, red and green lines, respectively

**Fig. 2:**
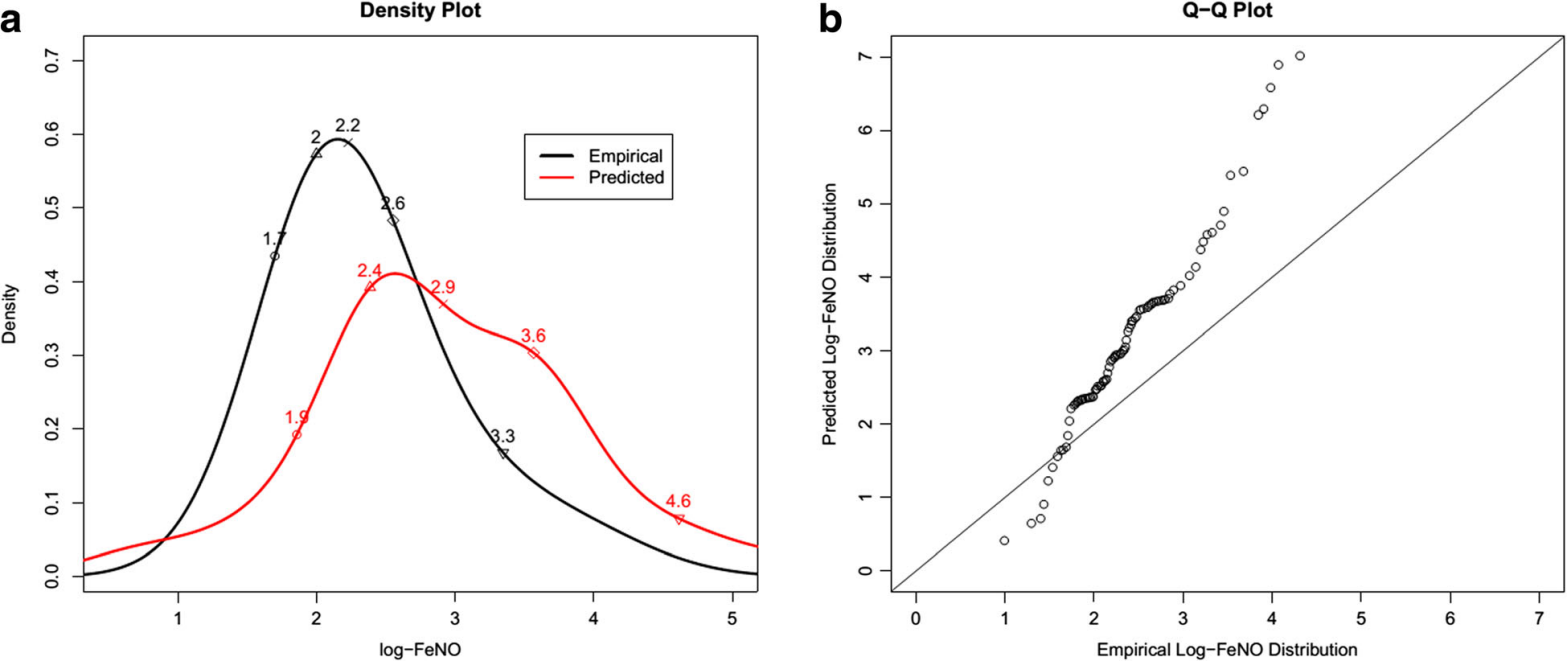
Empirical log-FeNO distribution among children without copy of the haplotype (black line) and its associated predicted distribution among children with two copies of haplotype, 10 μg/m^3^ increases in short-term PM_2.5_ exposure and 10% decreases in methylation level (Red line). Panel **a**) shows the density curves of both distributions and selected percentiles (10%, 30%, 50%, 70% and 90%), respectively. Panel **b**) is a QQ plot and plotted the quantiles of predicted distribution against those of empirical distribution

The quantile regression results are presented in conjunction with mean-based regression results in Figs. 1 and 2. Figure 1 shows joint effects on log-FeNO level at its mean and at selected quantiles, for each given copy number of *NOS2* haplotype, along with various combinations of decreases in iNOS promoter methylation and increases in short-term PM_2.5_ exposures from their average levels. Short-term PM_2.5_ exposure was more strongly associated with higher log-FeNO level when children carried at least one copy of *NOS2* haplotype and had lower percent iNOS promoter methylation. The observed phenomenon was not constant across the log-FeNO distribution, with larger and more significant joint effects at higher quantiles of FeNO.

Figure 2a demonstrates that short-term PM_2.5_ exposure, *NOS2* haplotype and iNOS promoter methylation jointly distort the log-FeNO distribution. Specifically, this figure compares the empirical log-FeNO distribution among children without a copy of the *NOS2* haplotype under observed short-term PM_2.5_ exposure and iNOS promoter methylation levels (black line) to their predicted log-FeNO distribution (red line). The predicted values are calculated when they all have two copies of *NOS2* haplotype and observed short-term PM_2.5_ exposure increased by 10 μg/m^3^ and observed iNOS promoter methylation levels decreased by 10 percentage points. In the official American Thoracic Society guidelines to the clinical interpretation of FeNO, for children with age < 12, FeNO >35 ppb indicates eosinophilic inflammation, and the use of corticosteroids is likely to be responsive for symptomatic individuals [3]. This figure shows that the proportion of children with FeNO >35 ppb is less than 10% among children with no copy of haplotype in the current study population. However, the proportion will increase to more than 20%, if the children have two copies of haplotype, 10 μg/m^3^ increase in short-term PM_2.5_ exposure and 10 percentage point decreased in methylation level. The Q-Q plot in Fig. 2b further shows that log-FeNO is distorted dramatically in the upper tails. These findings illustrate that the shift in mean log-FeNO distribution using standard means regression methods provides an incomplete summary that ignores critical features in these data.

Using quantile regression, we found significant modification of the joint effects of *NOS2* haplotype, iNOS promoter methylation level and short-term PM_2.5_ exposure on the FeNO distribution (Table 2). For example, asthma and race/ethnicity each significantly modified the joint effect on the 90th percentiles of the FeNO distribution (*p* = 0.03 and *p* = 0.01, respectively) and gender significantly modified the joint effect on the 10th, 50th and 70th percentiles of FeNO distribution (all p ≈ 0.01).This is in contrast to means regression, which did not detect these interactions of asthma, race/ethnicity and gender on mean log-FeNO (7 degree of freedom likelihood ratio test *p*-values are 0.63, 0.72 and 0.07, respectively). Children’s respiratory allergy status did not significantly modify the joint effects on either the mean or percentiles of log-FeNO (all *p* > 0.05).

**Table 2:** *P*-values from 7 degree freedom likelihood ratio tests evaluating effect modification by asthma, race/ethnicity, gender and allergy on the joint effects of *NOS2* haplotype, iNOS promoter methylation level and PM_2.5_

	*P*-Values					
	Mean	10%	30%	50%	70%	90%
Covariate						
Asthma (Yes vs No)	0.63	0.34	0.10	0.14	0.23	0.03
Race (Non-Hispanic White vs Hispanic White)	0.72	0.44	0.83	0.87	0.83	0.01
Gender (Male vs Female)	0.07	0.01	0.11	0.01	0.01	0.27
Allergy (Yes vs No)	0.98	0.34	0.38	0.89	0.92	0.64

**Fig. 3:**
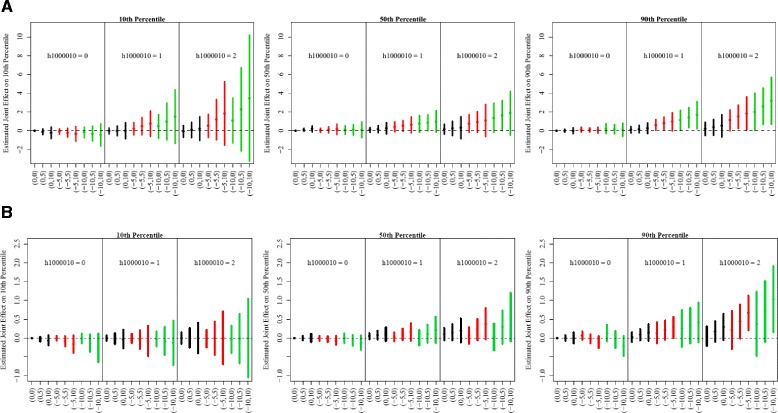
Estimated joint effects of *NOS2* H1 haplotype, iNOS methylation and 7-day average PM_2.5_ exposure across the selected quantiles of FeNO distribution and its mean by asthma status. In each panel, data are presented by number of H1 haplotype copy. The X-axis shows the combination of levels in methylation (first number in the bracket) and short-term PM_2.5_ exposure (second number in the bracket). Selected methylation levels are population average, 5% and 10% lower than averages, which are indexed by 0,-5 and −10, respectively. Selected PM_2.5_ exposure levels are population average, 5 μg/m^3^ and 10 μg/m^3^ higher PM_2.5_ exposure levels than average, which are indexed by 0, 5 and 10, respectively. The estimated joint effects when methylation levels are at population average, 5% and 10% lower than average are represented by black, red and green lines, respectively. Note: Y-axis limit for asthma group is wider than that for non-asthma group. **a** Asthma Group **b** Non-Asthma Group

**Fig. 4:**
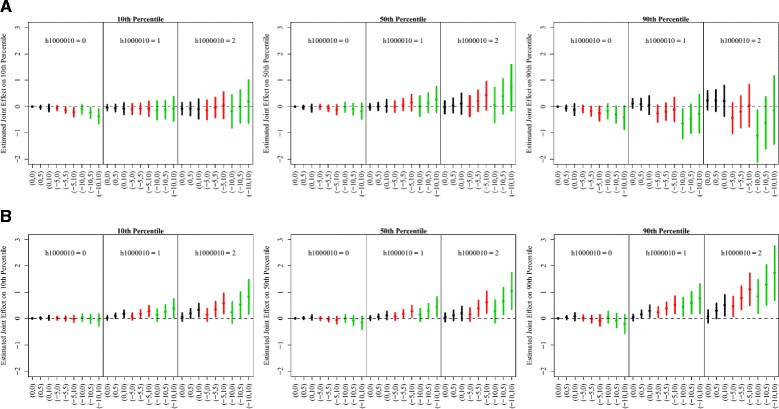
Estimated joint effects of *NOS2* H1 haplotype, iNOS methylation and 7-day average PM_2.5_ exposure across the selected quantiles of FeNO distribution and its mean by race. In each panel, data are presented by number of H1 haplotype copy. The X-axis shows the combination of levels in methylation (first number in the bracket) and short-term PM_2.5_ exposure (second number in the bracket). Selected methylation levels are population average, 5% and 10% lower than averages, which are indexed by 0,-5 and −10, respectively. Selected PM_2.5_ exposure levels are population average, 5 μg/m^3^ and 10 μg/m^3^ higher PM_2.5_ exposure levels than average, which are indexed by 0, 5 and 10, respectively. The estimated joint effects when methylation levels are at population average, 5% and 10% lower than average are represented by black, red and green lines, respectively. **a** Non-Hispanic White Group **b** Hispanic White Group

We found that the joint genetic, epigenetic and exposures effects among children with asthma were significantly larger than those among children without asthma, especially above the 90th percentiles of FeNO distribution (Fig. 3). Larger joint effects were observed among Hispanic white children across the FeNO distribution, especially on the 90th percentiles of FeNO, when compared with Non-Hispanic children (Fig. 4). Interestingly, we observed that among Non-Hispanic white children, lower iNOS promoter methylation was associated with lower log-FeNO at the 90th percentile for a given PM_2.5_ level and copy of *NOS2* haplotype. However, this association was not observed among Hispanic white children. In a similar joint effect figure by gender (Additional file 1: Figure S2), no simple joint effect pattern across the various combinations of PM_2.5_ exposure, iNOS promoter methylation level and copy of *NOS2* haplotype could be identified between male and female in Table 2.

Additional file 1: Figures S3, S4 and S5, similar to Fig. 2, depict the empirical and predicted log-FeNO distribution by asthma, race/ethnicity and gender, respectively. Additional file 1: Figures S3 and S4 show that the severe distortion in the upper tails of FeNO distribution are more dramatic among children with asthma and among the Hispanic white group for a 10 μg/m^3^ increases in short-term PM_2.5_ exposure and 10 percentage decreases in iNOS promoter methylation, and comparing 0 to 2 copies of *NOS2* haplotype. Additional file 1: Figure S4 shows that the same change in PM_2.5_, iNOS promoter methylation and *NOS2* haplotype markedly distorts the lower tail and middle part of the log-FeNO distribution in females but only distorts the upper tail in males.

Several models were fitted to assess the sensitivity of the results. The observed associations were robust to excluding subjects with exposure to second hand smoke (*N* = 33) and to adjustment for BMI, physical activity, and any asthma medication uses (data not shown). Besides *NOS2* promoter haplotype H1, we also explored the other common haplotype variants (H2-H4). Since H2 has inverse correlation with H1 (Spearman *r* = −0.43), its joint effects with PM_2.5_ and iNOS methylation for log-FeNO were significant, especially in the higher quantiles of FeNO, but opposite to what was found for H1 (data not shown). There were no significant interactive effects of iNOS methylation, PM_2.5_ exposure and H3 or H4 haplotypes across the log-FeNO distribution.

## Discussion

We discovered that the joint effects of short-term PM_2.5_ exposure, iNOS promoter methylation and *NOS2* haplotypes vary dramatically across the distribution of FeNO. These results went beyond the mean model by providing more insights to the joint effects of air pollution, genetic and epigenetic variants on the FeNO distribution. In this paper, our novel quantile regression analysis showed that the joint effects of short-term PM_2.5_ exposure, iNOS promoter methylation and *NOS2* haplotypes occurred predominantly in the upper tail of the FeNO distribution. This indicates that the genetic and epigenetic susceptibility of airway inflammation to short-term PM_2.5_ exposure is variable and greater among school children already having relatively high FeNO reflecting airway inflammation. Further, we discovered that the observed joint effects of short-term PM_2.5_ exposure, genetic and epigenetic variation in *NOS2* are greater among children with asthma and children identifying as Hispanic white who already have high FeNO level. This phenomenon was not evident from standard mean-based regression results due to the strong assumption of this method that effects are limited to shifts in the mean.

We infer that the observed larger associations of PM_2.5_, genetic and epigenetic variations in *NOS2* among the children already having high FeNO level are not fully attributable to the presence of asthma. In analyses of the subset of children without asthma, we found significantly stronger associations in the upper tail of FeNO distribution and the magnitudes of these associations were similar to those observed in the entire study population. The observed heterogeneous joint effect was also unlikely to be confounded by the level of asthma severity, which was not directly measured in CHS. CHS is a population-based cohort of school children. Therefore, asthma severity is low compared to typical clinical cohorts. Our results were robust in a sensitivity analysis that adjusted for asthma medication use, which can be treated as a surrogate for asthma severity.

These findings provide novel evidence of the importance of analyses that go beyond the standard approach estimating mean effects when evaluating the pathways for genetic and epigenetic variations and environmental exposure in phenotypic expression of respiratory disease. In the current epigenetic, genetic and air pollution health effects literature, mean regression is the dominant statistical approach due to its straightforward implementation and interpretation. However, this approach relies on the strong assumption that risk factors impact phenotype expression only through shifting its mean. Quantile regression can be considered a complement to mean regression which provides a more flexible way to understand the role of potential risk factors in the disease etiology and phenotype expression. In the past few years, the use of quantile regression in epidemiology and public health is gradually emerging. For example, Burgette et al. found that tobacco exposure depresses the 20th and 30th percentiles of birth weight more strongly among pregnant mothers with high level of lead in blood than those with low level [24]. Bind et al. showed that increases in particle number, PM_2.5_, black carbon and mass concentration were significantly associated with lower methylation in the lower tails of the *IFN-γ* and *ICAM-1* methylation distributions [25].

The observed novel varying effect of short-term PM_2.5_, genetic and epigenetic variations in iNOS promoter on FeNO among school children is biologically plausible. First, published studies have documented that short-term PM_2.5_ exposure and *NOS2* promoter haplotype are both associated with higher FeNO level [12, 26–31]. Secondly, lower iNOS promoter methylation has been associated with higher iNOS expression, and consequently increases in NO production and FeNO level [32]. The FeNO levels for children with low promoter methylation in *NOS2* are more likely to be in the upper tail of FeNO distribution. Thirdly, increases in short-term PM_2.5_ were more significantly associated with lower methylation in the lower tail of iNOS promoter methylation distribution. In other words, we hypothesize that higher short-term PM_2.5_ exposure could lead to sharper fall in iNOS promoter methylation among children with lower DNA methylation than those with comparatively high methylation. Therefore, it is plausible that the observed significant joint effects on the upper tail of FeNO distribution are attributable to the significant effect of PM_2.5_ on the lower tail of iNOS promoter methylation distribution, especially among children carrying this *NOS2* haplotype. Further studies are needed to fully evaluate the temporal patterns of exposure on iNOS DNA methylation and resultant changes in FeNO level and the influence of DNA-sequence variants in *NOS2* in such associations.

The strengths of the current study include the large, population-based cohort of school children, rigorous FeNO data quality control, and a thorough investigation of the joint effects of PM_2.5_, iNOS promoter methylation and *NOS2* haplotypes on FeNO using a cutting-edge statistical approach.

Results from our study should be interpreted in light of some limitations. The cross-sectional nature of the analysis precludes us from assessing the effects of some long term health confounders and temporal effects and addressing the causality. Our covariates were assessed by questionnaire, potentially introducing recall bias and misclassification. Because such misclassification usually biases the results towards null, it is unlikely to explain the observed findings. Moreover, the questionnaire items we employ are widely used in similar studies and treated as standardized core questions to estimate the prevalence of asthma status and current nose symptoms [33]. The findings may not be generalizable to other ethnic groups, as the study population was primarily non-Hispanic white and Hispanic children in southern California. Although the joint effects of PM_2.5_, iNOS promoter methylation and *NOS2* haplotypes on FeNO were also modified by gender, a clear illustration for this difference across various combinations of these exposures is still lacking and further study is warranted to better understand the role of gender in the observed joint effects on FeNO.

Although this manuscript primarily focus on understanding the joint effects of short-term PM_2.5_, genetic and epigenetic variants on FeNO, more complete investigation of other air pollutants, including the components of PM_2.5_, is warranted for future work. Meanwhile, genetic and epigenetic susceptibility of air inflammation to long-term air pollution exposure remains unknown and need to be answered in the future work.

## Conclusions

In summary, we found that school children with high FeNO have increased genetic and epigenetic susceptibility to short-term PM_2.5_ exposure. We also showed that standard mean regression failed to capture how PM_2.5_, iNOS promoter methylation and *NOS2* haplotypes jointly distort the FeNO distribution, primarily affecting the upper tail. Using quantile regression, we further discovered the phenomena that joint effects on the upper tail of the FeNO distribution are more significant among children with asthma or who are Hispanic white, which was also not found using mean-based regression. Quantile regression allows the estimation of associations across the response distribution, and could be used more widely, as either an alternative or a complement to mean regression approach for better understanding the pathway of environmental exposure, genetic and epigenetic factors in phenotype expression in future studies.

## Additional file

Additional file 1: Table S1. Joint effects of NOS2 H3 promoter haplotype, iNOS promoter methylation and 7-day average PM_2.5_ exposure on the distribution of FeNO. **Figure S1.** The variation of short-term PM_2.5_ exposure by town in the study cohort. (ANH = Anaheim; GLN = Glendora; LGB = Long Beach; MLM = Mira Loma; RIV = Riverside; SBB = Santa Barbara; SDE = San Dimas; UPL = Upland.). **Figure S2.** Estimated joint effects of *NOS2* H1 haplotype, iNOS methylation and 7-day average PM_2.5_ exposure across the selected quantiles of FeNO distribution and its mean by gender. In each panel, data are presented by number of H1 haplotype copy. **Figure S3.** Asthma specific empirical log-FeNO distributions among children without copy of the haplotype (Asthma: solid black line; Non-Asthma: dash black line) and their associated predicted distributions among children with two copies of haplotype, 10 μg/m^3^ increases in short-term PM_2.5_ exposure and 10% decreases in methylation level (Asthma: solid red line; Non-Asthma: dash black line). **Figure S4.** Race/Ethnicity specific empirical log-FeNO distributions among children without copy of the haplotype (White: solid black line; Hispanic: dash black line) and their associated predicted distributions among children with two copies of haplotype, 10 μg/m^3^ increases in short-term PM_2.5_exposure and 10% decreases in methylation level (White: solid red line; Hispanic: dash black line). **Figure S5.** Gender specific empirical log-FeNO distributions among children without copy of the haplotype (Male: solid black line; Female: dash black line) and their associated predicted distributions among children with two copies of haplotype, 10 μg/m^3^increases in short-term PM_2.5_ exposure and 10% decreases in methylation level (Male: solid red line; Female: dash black line). (445 KB)

## Abbreviations

ATS: American Thoracic Society
BMI: Body mass index
CHS: Southern California Children Health Study
FeNO: Fractional concentration of exhaled nitric oxide
iNOS: Inducible nitric oxide synthase
PM_2.5_: Particulate matter with aerodynamic diameter < =2.5 μm
QR: Quantile regression
SHS: Secondhand Tobacco Smoke
