# Supplementary material for: Genetic and epigenetic susceptibility of airway inflammation to PM2.5 in school children: new insights from quantile regression
Source: Environ Health. 2017 Aug 18;16:88. doi: 10.1186/s12940-017-0285-6 (PMC5563051; doi:10.1186/s12940-017-0285-6)
Supplement: Additional file 1: Table S1. — Joint effects of NOS2 H3 promoter haplotype, iNOS promoter methylation and 7-day average PM2.5 exposure on the distribution of FeNO. Figure S1. The variation of short-term PM2.5 exposure by town in the study cohort. (ANH = Anaheim; GLN = Glendora; LGB = Long Beach; MLM = Mira Loma; RIV = Riverside; SBB = Santa Barbara; SDE = San Dimas; UPL = Upland.). Figure S2. Estimated joint effects of NOS2 H1 haplotype, iNOS methylation and 7-day average PM2.5 exposure across the selected quantiles of FeNO distribution and its mean by gender. In each panel, data are presented by number of H1 haplotype copy. Figure S3. Asthma specific empirical log-FeNO distributions among children without copy of the haplotype (Asthma: solid black line; Non-Asthma: dash black line) and their associated predicted distributions among children with two copies of haplotype, 10 μg/m3 increases in short-term PM2.5 exposure and 10% decreases in methylation level (Asthma: solid red line; Non-Asthma: dash black line). Figure S4. Race/Ethnicity specific empirical log-FeNO distributions among children without copy of the haplotype (White: solid black line; Hispanic: dash black line) and their associated predicted distributions among children with two copies of haplotype, 10 μg/m3 increases in short-term PM2.5exposure and 10% decreases in methylation level (White: solid red line; Hispanic: dash black line). Figure S5. Gender specific empirical log-FeNO distributions among children without copy of the haplotype (Male: solid black line; Female: dash black line) and their associated predicted distributions among children with two copies of haplotype, 10 μg/m3increases in short-term PM2.5 exposure and 10% decreases in methylation level (Male: solid red line; Female: dash black line). (445 KB) [file 12940_2017_285_MOESM1_ESM.docx]

**Supplementary Material**

**Genetic and Epigenetic Susceptibility of Airway Inflammation to PM_2.5_ in School Children: New Insights from Quantile Regression**

**Authors:** Yue Zhang^1,2,3^, Muhammad T. Salam^4,5^, Kiros Berhane^4^, Sandrah P. Eckel^4^, Edward B. Rappaport^4^, William S. Linn^4^, Rima Habre^4^, Theresa M Bastain^4^, Frank D. Gilliland^4^

**Affiliations:**^1^ Department of Internal Medicine, University of Utah, Salt Lake City, UT, USA;

^2^ Department of Family and Preventive Medicine, University of Utah, Salt Lake City, UT, USA;

^3^ Veteran Affairs Salt Lake City Health Care System, Salt Lake City, UT, USA;

^4^ Department of Preventive Medicine, University of Southern California, Los Angeles, CA, USA;

^5^ Department of Psychiatry, Kern Medical, Bakersfield, CA, USA

**Correspondence:** Yue Zhang, Division of Epidemiology**,** Department of Internal Medicine**,** University of Utah**,** 295 Chipeta Way**,** Salt Lake City, UT, 84018**,** E-mail: [zhang.yue@hsc.utah.edu](mailto:zhang.yue@hsc.utah.edu).

**Summary:** School-aged children with higher FeNO have greater genetic and epigenetic susceptibility to PM_2.5_, highlighting the importance of investigating effects across the entire distribution of FeNO.

**Table of Contents**

**Methods:**

**Table S1:** Joint effects of NOS2 H3 promoter haplotype, iNOS promoter methylation and 7-day average PM_2.5_ exposure on the distribution of FeNO.

**Figure S1**: Estimated joint effects of *NOS2* H1 haplotype, iNOS methylation and 7-day average PM_2.5_ exposure across the selected quantiles of FeNO distribution and its mean by gender. In each panel, data are presented by number of H1 haplotype copy. The X-axis shows the combination of levels in methylation (first number in the bracket) and short-term PM_2.5_ exposure (second number in the bracket). Selected methylation levels are population average, 5% and 10% lower than average, which are indexed by 0,-5 and -10, respectively. Selected PM_2.5_ exposure levels are population average, 5μg/m^3^ and 10μg/m^3^ higher PM_2.5_ exposure levels than average, which are indexed by 0, 5 and 10, respectively. The estimated joint effects when methylation levels are at population average, 5% and 10% lower than average are represented by black, red and green lines, respectively.

**Figure S2**: Asthma specific empirical log-FeNO distributions among children without copy of the haplotype (Asthma: solid black line; Non-Asthma: dash black line) and their associated predicted distributions among children with two copies of haplotype, 10μg/m^3^ increases in short-term PM2.5 exposure and 10% decreases in methylation level (Asthma: solid red line; Non-Asthma: dash black line). Panel A) showed the asthma specific density curves of both distributions and selected percentiles (10%, 30%, 50%, 70% and 90%), respectively. Panel B) presented asthma specific QQ plots which plotted the quantiles of predicted distribution against those of empirical distribution.

**Figure S3**: Race/Ethnicity specific empirical log-FeNO distributions among children without copy of the haplotype (White: solid black line; Hispanic: dash black line) and their associated predicted distributions among children with two copies of haplotype, 10μg/m^3^ increases in short-term PM_2.5_ exposure and 10% decreases in methylation level (White: solid red line; Hispanic: dash black line). Panel A) showed the asthma specific density curves of both distributions and selected percentiles (10%, 30%, 50%, 70% and 90%), respectively. Panel B) presented race/ethnicity specific QQ plots which plotted the quantiles of predicted distribution against those of empirical distribution.

**Figure S4**: Gender specific empirical log-FeNO distributions among children without copy of the haplotype (Male: solid black line; Female: dash black line) and their associated predicted distributions among children with two copies of haplotype, 10μg/m^3^ increases in short-term PM_2.5_ exposure and 10% decreases in methylation level (Male: solid red line; Female: dash black line). Panel A) showed the asthma specific density curves of both distributions and selected percentiles (10%, 30%, 50%, 70% and 90%), respectively. Panel B) presented gender specific QQ plots which plotted the quantiles of predicted distribution against those of empirical distribution.

**Table S1: Joint effects of NOS2 H1 promoter haplotype, iNOS promoter methylation and 7-day average PM_2.5_ exposure on the distribution of FeNO**

|  | **Estimated Effects (SE) on Mean and Different Percentiles of log-transformed FeNO Distribution** | | | | | |
| --- | --- | --- | --- | --- | --- | --- |
| **Factors** | **Mean** | **10%** | **30%** | **50%** | **70%** | **90%** |
| PM_2.5_ Exposure (PM) | 0.01(0.02) | -0.02(0.02) | -0.01(0.02) | 0.01(0.02) | 0.05(0.03) | 0.03(0.04) |
| H1 haplotype (H1) | 0.03(0.03) | -0.01(0.03) | 0.01(0.03) | 0.04(0.03) | 0.09(0.05) | 0.04(0.06) |
| iNOS methylation (M) | -0.02(0.03) | 0.01(0.02) | -0.03(0.02) | 0.00(0.03) | 0.00(0.04) | -0.07(0.05) |
| H1×PM | 0.04(0.02)* | 0.02(0.02) | 0.02(0.02) | 0.02(0.02) | 0.03(0.03) | 0.09(0.03)* |
| M×PM | 0.06(0.02)** | 0.05(0.01)** | 0.02(0.01) | 0.03(0.02) | 0.07(0.03)** | 0.09(0.03)** |
| H1×M | -0.02(0.04) | -0.01(0.03) | -0.01(0.03) | -0.01(0.04) | -0.05(0.06) | -0.07(0.06) |
| H1×M× PM | -0.09(0.02)** | -0.03(0.02) | -0.03(0.02) | -0.08(0.02)** | -0.09(0.04)* | -0.09(0.04)* |

*: 0.01<P-value <0.05; **: P-value<0.01;

**Figure S1:** The variation of short-term PM_2.5_ exposure by town in the study cohort. (ANH=Anaheim; GLN=Glendora; LGB=Long Beach; MLM=Mira Loma; RIV=Riverside; SBB=Santa Barbara; SDE=San Dimas; UPL=Upland.)

**A: Male Group:**

**
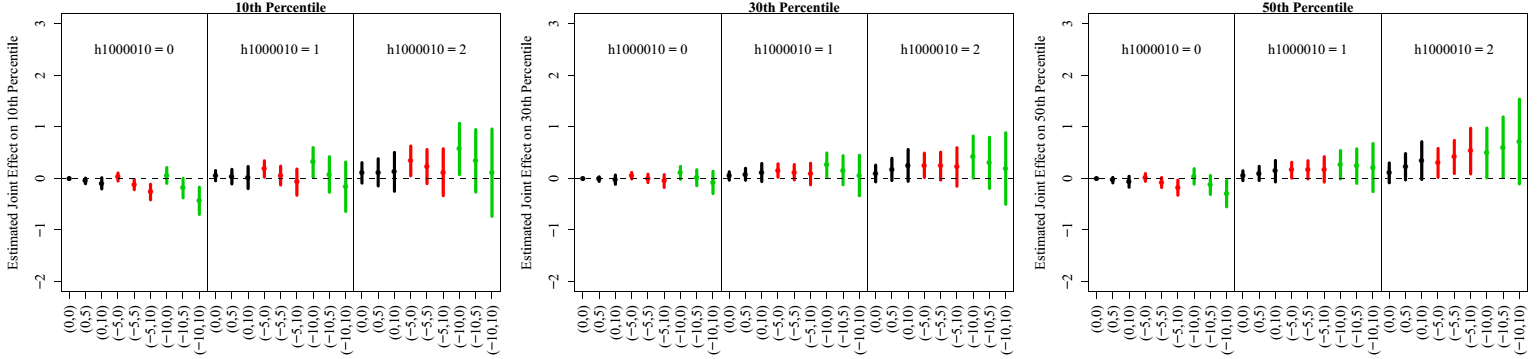
**

**B: Female Group:**

**
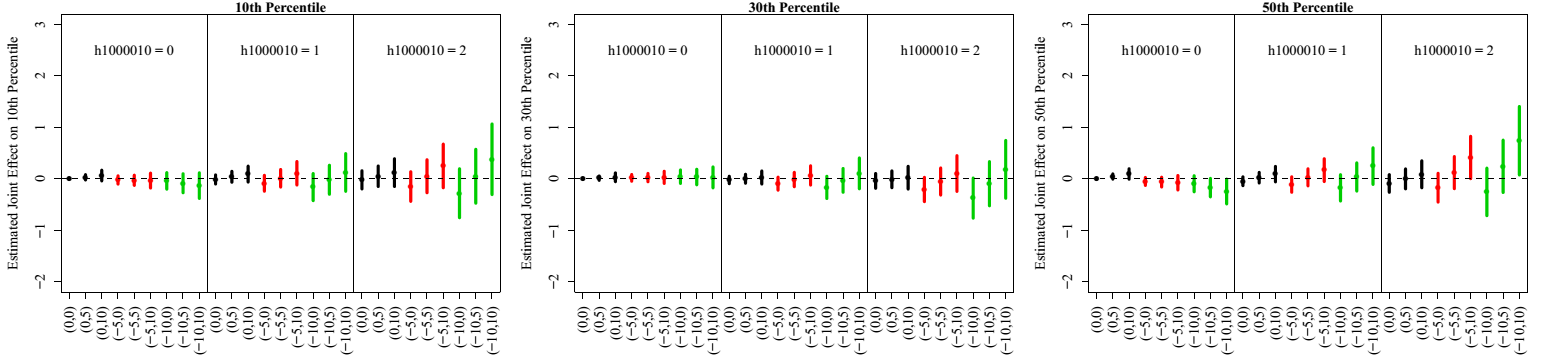
**

**Figure S2:** Estimated joint effects of *NOS2* H1 haplotype, iNOS methylation and 7-day average PM_2.5_ exposure across the selected quantiles of FeNO distribution and its mean by gender. In each panel, data are presented by number of H1 haplotype copy. The X-axis shows the combination of levels in methylation (first number in the bracket) and short-term PM_2.5_ exposure (second number in the bracket). Selected methylation levels are population average, 5% and 10% lower than average, which are indexed by 0,-5 and -10, respectively. Selected PM_2.5_ exposure levels are population average, 5μg/m^3^ and 10μg/m^3^ higher PM_2.5_ exposure levels than average, which are indexed by 0, 5 and 10, respectively. The estimated joint effects when methylation levels are at population average, 5% and 10% lower than average are represented by black, red and green lines, respectively.

**
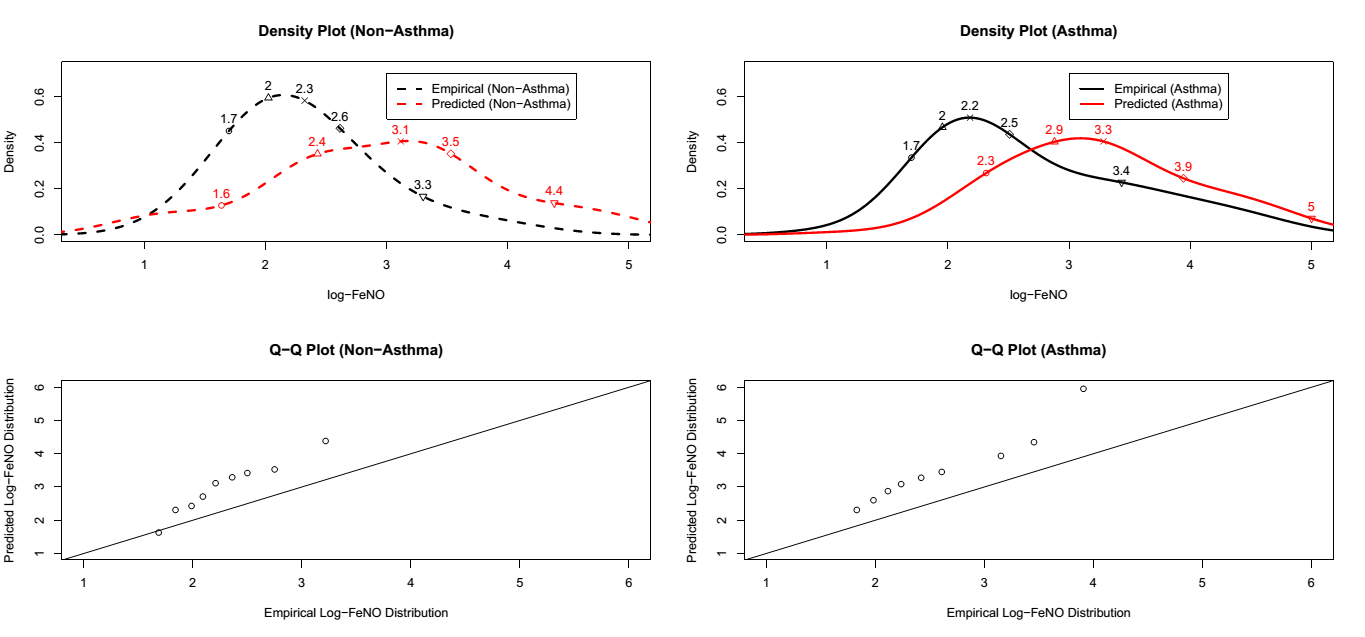
**

**B)**

**A)**

**Figure S3:** Asthma specific empirical log-FeNO distributions among children without copy of the haplotype (Asthma: solid black line; Non-Asthma: dash black line) and their associated predicted distributions among children with two copies of haplotype, 10μg/m^3^ increases in short-term PM_2.5_ exposure and 10% decreases in methylation level (Asthma: solid red line; Non-Asthma: dash black line). Panel A) showed the asthma specific density curves of both distributions and selected percentiles (10%, 30%, 50%, 70% and 90%), respectively. Panel B) presented asthma specific QQ plots which plotted the quantiles of predicted distribution against those of empirical distribution.

**
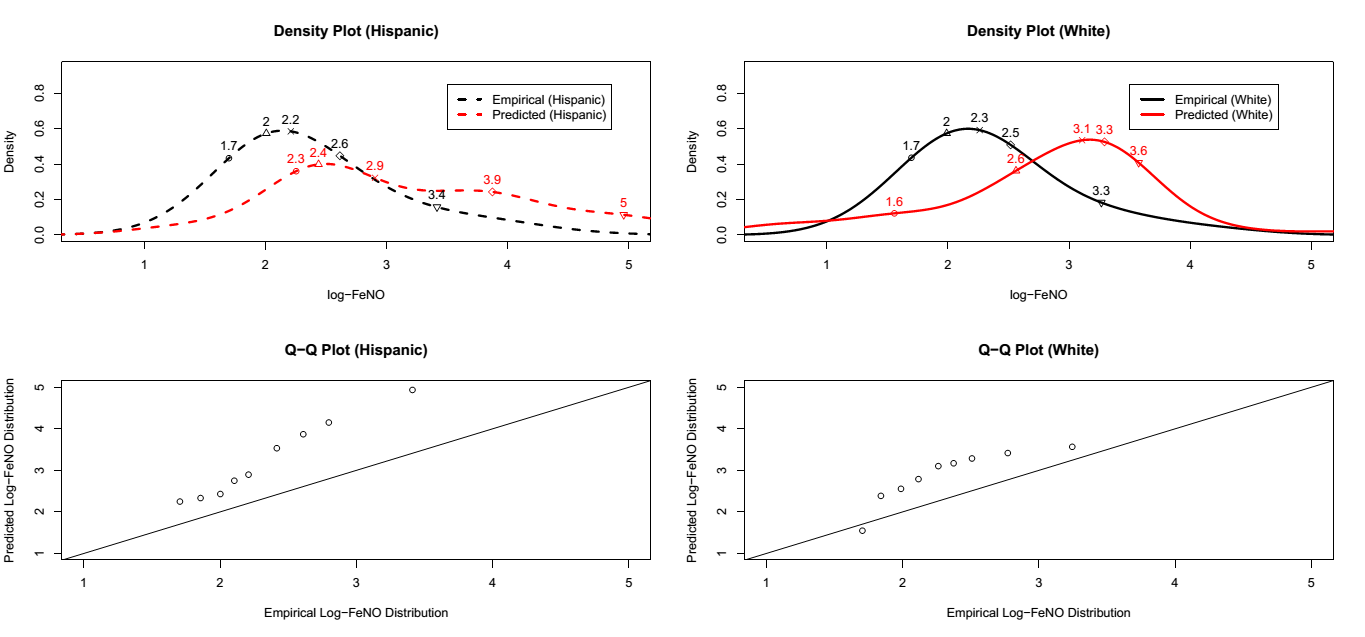
**

**B)**

**A)**

**Figure S4:** Race/Ethnicity specific empirical log-FeNO distributions among children without copy of the haplotype (White: solid black line; Hispanic: dash black line) and their associated predicted distributions among children with two copies of haplotype, 10μg/m^3^ increases in short-term PM_2.5_ exposure and 10% decreases in methylation level (White: solid red line; Hispanic: dash black line). Panel A) showed the asthma specific density curves of both distributions and selected percentiles (10%, 30%, 50%, 70% and 90%), respectively. Panel B) presented race/ethnicity specific QQ plots which plotted the quantiles of predicted distribution against those of empirical distribution.

**
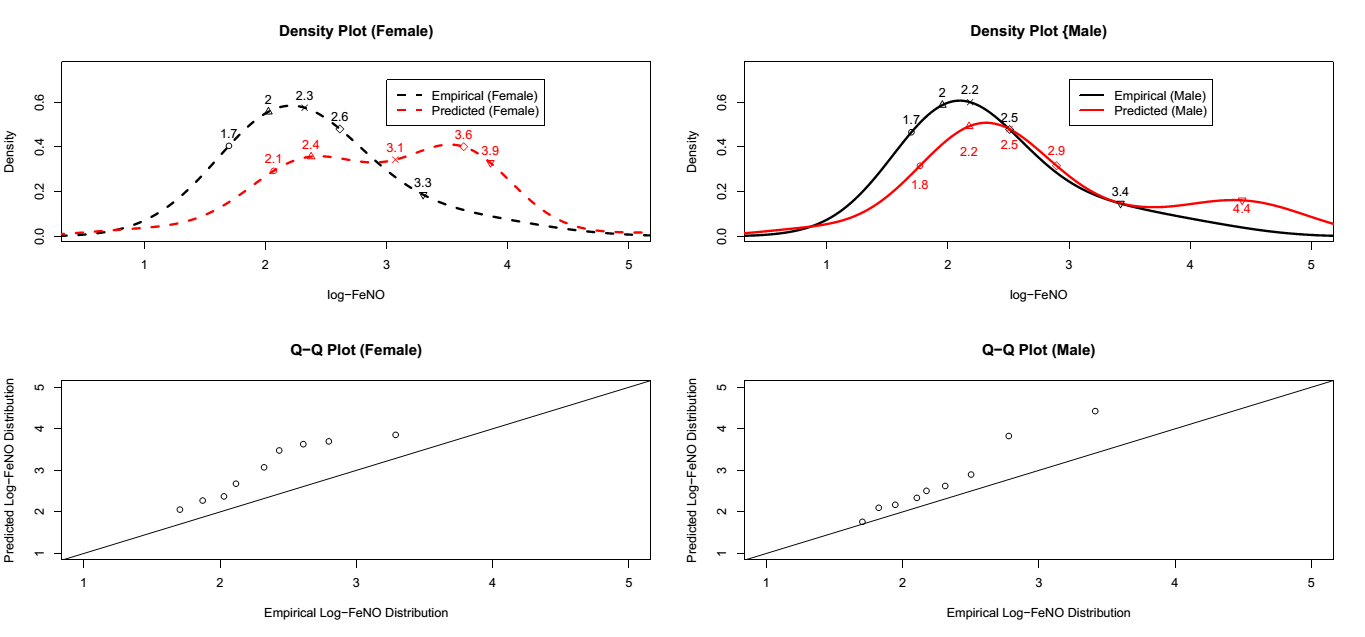
**

**A)**

**B)**

**Figure S5:** Gender specific empirical log-FeNO distributions among children without copy of the haplotype (Male: solid black line; Female: dash black line) and their associated predicted distributions among children with two copies of haplotype, 10μg/m^3^ increases in short-term PM_2.5_ exposure and 10% decreases in methylation level (Male: solid red line; Female: dash black line). Panel A) showed the asthma specific density curves of both distributions and selected percentiles (10%, 30%, 50%, 70% and 90%), respectively. Panel B) presented gender specific QQ plots which plotted the quantiles of predicted distribution against those of empirical distribution.
